# Ectogestation ethics: The implications of artificially extending gestation for viability, newborn resuscitation and abortion

**DOI:** 10.1111/bioe.12682

**Published:** 2019-11-07

**Authors:** Lydia Di Stefano, Catherine Mills, Andrew Watkins, Dominic Wilkinson

**Affiliations:** ^1^ Faculty of Medicine, Nursing and Health Sciences Monash University Melbourne Australia; ^2^ Oxford Uehiro Centre for Practical Ethics Faculty of Philosophy University of Oxford Oxford United Kingdom; ^3^ Neonatal Unit Mercy Hospital for Women Melbourne Australia; ^4^ Newborn Care John Radcliffe Hospital Oxford United Kingdom; ^5^ Murdoch Children’s Research Institute Melbourne Australia

**Keywords:** abortion, artificial womb, ectogenesis, ectogestation, infant, medical ethics, neonatal resuscitation, premature, termination of pregnancy

## Abstract

Recent animal research suggests that it may soon be possible to support the human fetus in an artificial uterine environment for part of a pregnancy. A technique of extending gestation in this way (“ectogestation”) could be offered to parents of extremely premature infants (EPIs) to improve outcomes for their child. The use of artificial uteruses for ectogestation could generate ethical questions because of the technology’s potential impact on the point of “viability”—loosely defined as the stage of pregnancy beyond which the fetus may survive external to the womb. Several medical decisions during the perinatal period are based on the gestation at which infants are considered viable, for example decisions about newborn resuscitation and abortion, and ectogestation has the potential to impact on these. Despite these possible implications, there is little existing evidence or analysis of how this technology would affect medical practice. In this paper, we combine empirical data with ethical analysis. We report a survey of 91 practicing Australian obstetricians and neonatologists; we aimed to assess their conceptual understanding of “viability,” and what ethical consequences they envisage arising from improved survival of EPIs. We also assess what the ethical implications of extending gestation should be for newborn and obstetric care. We analyze the concept of viability and argue that while ectogestation might have implications for the permissibility of neonatal life‐prolonging treatment at extremely early gestation, it should not necessarily have implications for abortion policy. We compare our ethical findings with the results of the survey.

## 1 ECTOGESTATION AND VIABILITY

The idea of an artificial womb has been extensively explored over the last century in science fiction, literature, and film.1Smajdor, A. (2007). The moral imperative for ectogenesis. *Cambridge Quarterly of Healthcare Ethics, 16*(3), 336–345. Philosophers and ethicists have also written on this topic, with articles in both academic literature and the mainstream press exploring the ethics of gestating embryos and fetuses outside the female body.2Ibid; Cannold, L. (1995). Women, ectogenesis and ethical theory. *Journal of Applied Philosophy, 12*(1), 55–64; Aristarkhova, I. (2005). Ectogenesis and mother as machine. *Body & Society, 11*(3), 43–59. https://doi.org/10.1177/1357034X05056190; Kendal, E. (2017). The perfect womb: Promoting equality of (fetal) opportunity. *Journal of Bioethical Inquiry, 14*(2), 185–194. https://doi.org/10.1007/s11673-017-9775-z; Steiger, E. (2010). Not of woman born: How ectogenesis will change the way we view viability, birth, and the status of the unborn. *Journal of Law and Health, 2*3, 143–171; Murphy, J. S. (1989). Is pregnancy necessary? Feminist concerns about ectogenesis. *Hypatia, 4*(3), 66–84; Lupton, M. L. (1998). Artificial reproduction and the family of the future. *Medicine and Law, 17*(1), 93–111; Lupton, M. L. (1997). Artificial wombs: Medical miracle, legal nightmare. *Medicine and Law, 16*(3), 621–633; Yuko, E. (2017, May 8). Weighing the ethics of artificial wombs. *The New York Times.* Retrieved from: https://www.nytimes.com/2017/05/08/health/artificial-wombs-ethics.html; Sedgwick, H. (2017, Sep 4). Artificial wombs could soon be a reality. What will this mean for women? *The Guardian.* Retrieved from: https://www.theguardian.com/lifeandstyle/2017/sep/04/artifical-womb-women-ectogenesis-baby-fertility.


However, discussion has taken a more practical turn following the publication in 2017 of a report on a technique for supporting extremely premature newborn lambs for up to 4 weeks in a “Biobag.”3Partridge, E. A., Davey, M. G., Hornick, M. A., McGovern, P. E., Mejaddam, A. Y., Vrecenak, J. D., . . . Flake, A. W. (2017). An extra‐uterine system to physiologically support the extreme premature lamb. *Nature Communications, 8*, 15112. https://doi.org/10.1038/ncomms15112. In the landmark study from Philadelphia, lambs were delivered by cesarean section at a level of lung maturity equivalent to humans at 23 weeks gestation (17 weeks early). Blood vessels in the umbilical cord were connected rapidly to a low resistance oxygenator circuit, which provided artificial intravenous nutrition. The lambs were supported within a sealed fluid‐filled bag, the artificial amniotic fluid continuously exchanged to prevent infection. Eight of 13 lambs were sustained for 20–28 days using this system.4Researchers called the technique “EXTra‐uterine Environment for Neonatal Development” (EXTEND) due to its aim to extend gestation. Partridge, E. A., Davey, M. G., Hornick, M. A., & Flake, A. W. (2017). An extrauterine environment for neonatal development: Extending fetal physiology beyond the womb. *Seminars in Fetal and Neonatal Medicine, 22*(6), 404–409.


While the technology for “full ectogenesis” (undertaking the full pregnancy outside the human body) appears to be a long way off, the Philadelphia study suggests that it may soon be possible to support human fetuses for part of a pregnancy. This technique is sometimes referred to as partial ectogenesis, but we will use the term “ectogestation,” since this refers more accurately to a period of external gestation rather than external creation.

It has been proposed that ectogestation could be offered to extremely premature infants (EPIs) to improve outcomes.5Ibid. Ectogestation would represent a form of potentially life‐prolonging treatment. Some of the most premature infants have high rates of mortality and morbidity with existing forms of intensive care.6Patel, R. M. (2016). Short‐ and long‐term outcomes for extremely preterm infants. *American Journal of Perinatology, 33*(3), 318–328. https://doi.org/10.1055/s-0035-1571202. Their immature lungs are easily damaged by mechanical ventilation, and gas exchange may be difficult or impossible.7Bird, S. D. (2017). Artificial placenta: Analysis of recent progress. *European Journal of Obstetrics & Gynecology Reproductive Biology, 208*, 61–70. https://doi.org/10.1016/j.ejogrb.2016.11.005. If a baby who is about to be born extremely prematurely (because of preterm labor or severe maternal illness) could be transferred into an artificial womb for a period of time, this may allow sufficient growth and maturity to substantially improve their health outcomes.

The development of ectogestation would have potential ethical implications for both obstetrics and neonatology. These include questions arising directly from the implementation of ectogestation—for example, regarding how the therapy should be evaluated, when or if randomized trials involving humans would be ethical, when the benefits would outweigh the risks of such a therapy, and the implications for resource allocation.8Romanis, E. C. (2018). Artificial womb technology and the frontiers of human reproduction: Conceptual differences and potential implications. *Journal of Medical Ethics, 44*(11), 751–755. https://doi.org/10.1136/medethics-2018-104910; Mercurio, M. R. (2018). The EXTEND system for extrauterine support of extremely premature neonates: Opportunity and caution. *Pediatric Research, 84*(6), 795–796. https://doi.org/10.1038/s41390-018-0198-2; Cohen, I. G. (2017). Artificial wombs and abortion rights. *Hastings Center Report, 47*(4), inside back cover. https://doi.org/10.1002/hast.730; Wilkinson, D., & Di Stefano, L. (in press). Artificial gestation. In E. Boyle & J. Cusack (Eds.), *Hot topics and controversies in neonatal care*. Springer.


However, the development of ectogestation could also generate ethical questions through its potential impact on viability. Viability can loosely be described as the ability of a fetus or infant to survive independently of its pregnant mother, although the precise definition is contested.9Fost, N., Chudwin, D., & Wikler, D. (1980). The limited moral significance of ‘fetal viability’. *Hastings Center Report, 10*(6), 10–13. https://doi.org/10.2307/3560289; Gert, H. J. (1995). Viability. *International Journal of Philosophical Studies, 3*(1), 133–142. https://doi.org/10.1080/09672559508570807; Gillon, R. (2001). Is there a ‘new ethics of abortion’? *Journal of Medical Ethics, 27*(Suppl 2), ii5; Glass, H. C., Costarino, A. T., Stayer, S. A., Brett, C. M., Cladis, F., & Davis, P. J. (2015). Outcomes for extremely premature infants. *Anesthesia & Analgesia, 120*(6), 1337–1351. https://doi.org/10.1213/ANE.0000000000000705; Jensen, D. (2015). Birth, meaningful viability and abortion. *Journal of Medical Ethics, 41*(6), 460–463; Erdman, J. N. (2017). Theorizing time in abortion law and human rights. *Health and Human Rights, 19*(1), 29–40; Pignotti, M. S. (2010). The definition of human viability: A historical perspective. *Acta Paediatrica, 99*(1), 33–36. https://doi.org/10.1111/j.1651-2227.2009.01524.x; Viability appears to be distinct from the question of whether the fetus is a “person.” We will discuss in Section 3 the possibility that viability confers moral status; however, for the most part in this paper we will set aside questions relating to fetal personhood. We will return to the definition in Section 3, but the basic idea is that prior to a certain point in pregnancy fetuses are not yet viable as they would not survive if they were delivered. However, beyond the point of viability, fetuses may be liveborn and may survive.10We will focus in this paper on fetuses/preterm infants without other major morbidities. Illness or abnormality may affect the chance of survival of the fetus and hence their “viability.” We will set aside the question of provision of intensive care and the option of abortion in the setting of fetal abnormality. Many medical decisions during the perinatal period are based on the gestation at which infants are considered viable. For example, resuscitation and intensive care are considered if newborn infants are viable, whereas these are usually withheld if the newborn is not yet viable. In many jurisdictions, abortion is permitted in the first half of pregnancy, but termination of pregnancy is not legally permitted if the fetus is considered viable.11Wood, M. A. H., & Hawkins, L. B. (1980). State regulation of late abortion and the physician's duty of care to the viable fetus. *Missouri Law Review, 45*, 394–422; De Crespigny, L. J., & Savulescu, J. (2004). Abortion: Time to clarify Australia's confusing laws. *Medical Journal of Australia, 181*(4), 201–203; Cohen, I. G., & Sayeed, S. (2011). Fetal pain, abortion, viability, and the constitution. *Journal of Law, Medicine & Ethics, 39*(2), 235–242. https://doi.org/10.1111/j.1748-720X.2011.00592.x; Han, L., Rodriguez, M. I., & Caughey, A. B. (2018). Blurred lines: Disentangling the concept of fetal viability from abortion law. *Women's Health Issues, 28*(4), 287–288. https://doi.org/10.1016/j.whi.2018.02.006. It is worth noting that other jurisdictions do not place normative weight on viability. They may restrict legal abortions at an earlier gestation. For example, in a number of European countries abortion is permitted on maternal request or for socio‐economic reasons only until 12 weeks (athough it may be permitted later where there is evidence of serious fetal abnormality). Levels, M., Sluiter, R., & Need, A. (2014). A review of abortion laws in Western‐European countries. A cross‐national comparison of legal developments between 1960 and 2010. *Health Policy, 118*(1), 95–104. It is beyond the scope of this paper to discuss the ethical reasons behind non‐viability‐related gestation cut‐offs in abortion policy. (Alternatively, abortion may be restricted beyond viability, for example, it might be permitted only in the presence of severe medical conditions in the fetus or pregnant mother.)

Although the researchers who undertook the Philadelphia study were at pains to emphasize that their aims were not to alter the borderline of viability,12Partridge et al., op. cit. note 3. it appears plausible that such a technology, if successfully translated to humans, could make it possible for EPIs to survive who could not previously have been saved. If ectogestation facilitated the survival of more immature EPIs, it could shift perceptions regarding the point of viability and therefore have potential implications for both newborn care and abortion.13Cohen, op. cit. note 8; Han et al., op. cit. note 11; Chervenak, F. A., McCullough, L. B., & Levene, M. I. (2007). An ethically justified, clinically comprehensive approach to peri‐viability: Gynaecological, obstetric, perinatal and neonatal dimensions. *Journal of Obstetrics and Gynaecology, 27*(1), 3–7. https://doi.org/10.1080/01443610601133605; Gillam, L., Wilkinson, D., Xafis, V., & Isaacs, D. (2017). Decision‐making at the borderline of viability: Who should decide and on what basis? *Journal of Paediatrics and Child Health, 53*(2), 105–111. https://doi.org/10.1111/jpc.13423.


While ectogestation draws attention to questions relating to changes in the point of viability, they are not unique to this technology. Current techniques for neonatal intensive care including developments in mechanical ventilation, exogenous surfactant and the routine administration of antenatal steroids have considerably improved outcomes for extremely premature infants since the 1980s.14Glass et al., op. cit. note 9; Santhakumaran, S., Statnikov, Y., Gray, D., Battersby, C., Ashby, D., Modi, N., & Medicines for Neonates Investigator Group. (2017). Survival of very preterm infants admitted to neonatal care in England 2008‐2014: Time trends and regional variation. *Archives of Disease in Childhood. Fetal and Neonatal Edition, 103*(3), F208‐F215. https://doi.org/10.1136/archdischild-2017-312748; Moore, T., Hennessy, E. M., Myles, J., Johnson, S. J., Draper, E. S., Costeloe, K. L., & Marlow, N. (2012). Neurological and developmental outcome in extremely preterm children born in England in 1995 and 2006: The EPICure studies. *BMJ, 345*, e7961. https://doi.org/10.1136/bmj.e7961; Costeloe, K., Hennessy, E., Gibson, A. T., Marlow, N., & Wilkinson, A. R. (2000). The EPICure study: Outcomes to discharge from hospital for infants born at the threshold of viability. *Pediatrics, 106*(4), 659–671. Whereas survival was rare prior to 24 weeks gestation in the 1990s15Costeloe et al., op. cit. note 14., some centers now report survival rates of 50% or greater at 22 weeks gestation for infants admitted to intensive care.16Mehler, K., Oberthuer, A., Keller, T., Becker, I., Valter, M., Roth, B., & Kribs, A. (2016). Survival among infants born at 22 or 23 weeks’ gestation following active prenatal and postnatal care. *JAMA Pediatrics, 170*(7), 671–677. https://doi.org/10.1001/jamapediatrics; Norman, M., Hallberg, B., Abrahamsson, T., Björklund, L. J., Domellöf, M., Farooqi, A., … Ingemansson, F. (2019). Association between year of birth and 1‐year survival among extremely preterm infants in Sweden during 2004‐2007 and 2014‐2016. *JAMA: The Journal of the American Medical Association, 321*(12), 1188–1199; Kono, Y., Yonemoto, N., Nakanishi, H., Kusuda, S., & Fujimura, M. (2018). Changes in survival and neurodevelopmental outcomes of infants born at < 25 weeks’ gestation: A retrospective observational study in tertiary centres in Japan. *BMJ Paediatrics Open, 2*(1), e000211. Have such improvements led to changes in perceptions of viability and the thresholds for resuscitation/provision of intensive care17In this paper, we will sometimes use the term “resuscitation” as short‐hand for “resuscitation and provision of intensive care/life‐prolonging treatment.” However, we do not mean to restrict discussion to simply initial delivery room management of extreme preterm infants. or abortion?

Little is known about how specialist professionals who provide medical care for pregnant women and for EPIs understand the concept and ethical implications of viability. A 2011 qualitative study surveyed New York obstetricians and neonatologists in administrative roles.18Ramsay, S. M., & Santella, R. M. (2011). The definition of life: A survey of obstetricians and neonatologists in New York City hospitals regarding extremely premature births. *Maternal and Child Health Journal, 15*(4), 446–452. The majority of physicians (81%) considered an infant born ≥23 weeks gestation viable, and 97% of neonatologists said they always resuscitate such infants.

Other studies have explored how health professionals view the link between viability and abortion, including a 2016 survey, which included individuals who manage or provide counseling to prenatal patients in Canada.19Hull, D., Davies, G., & Armour, C. M. (2016). Survey of the definition of fetal viability and the availability, indications, and decision making processes for post‐viability termination of pregnancy for fetal abnormalities and health conditions in Canada. *Journal of Genetic Counseling, 25*(3), 543–551. https://doi.org/10.1007/s10897-015-9907-8. Surveyed counselors were most likely to consider 24 weeks the point of fetal viability for post‐viability termination of pregnancy. Another study asked obstetricians in eight European countries about their view on late abortion.20Habiba, M., Frè, M. D., Taylor, D., Arnaud, C., Bleker, O., Lingman, G., … Viafora, C. (2009). Late termination of pregnancy: a comparison of obstetricians’ experience in eight European countries. *BJOG: An International Journal of Obstetrics & Gynaecology, 116*(10), 1340–1349. https://doi.org/10.1111/j.1471-0528.2009.02228.x. In each country, the obstetricians reported sometimes performing abortion after 23 weeks gestation. The majority of surveyed obstetricians in seven of the countries felt that there should be no change to abortion policy in their country.21There are multiple other papers that discuss health professionals’ views on viability and fetal anomaly. These have not been included as the focus of this paper is on morphologically normal fetuses and the evolution of viability during pregnancy.


In Section 2 of the paper, we report a detailed survey of obstetric and neonatal medical specialists, those most likely to be aware of changes in the outcome of EPIs, and to be regularly encountering questions relating to neonatal resuscitation and abortion. The aim is to investigate professionals’ conceptual understanding of “viability,” and what ethical consequences they identify arising from improved survival of EPIs using existing or future technology.

## 2 EMPIRICAL SURVEY

### 2.1 Methods

#### 2.1.1 Participants and procedure

An online survey was developed to determine the views of specialist doctors on questions relating to medical treatment at the borderline of viability. Participants were asked to consider several scenarios relating to neonatal and obstetric management of preterm labor and EPIs in the light of current and future technologies.

Before distribution, the survey was piloted on a group of medical students and doctors in obstetric/neonatal practice. Suggestions were incorporated into the final survey.

The study participants consisted of specialist doctors (consultants or registrars/fellows in obstetrics and neonatology) practicing in Victoria, Australia. Victoria has a population of 6.4 million, with approximately 82,000 births per year.22Australian Bureau of Statistics. (2018). Victoria (STE). Retrieved from https://itt.abs.gov.au/itt/r.jsp?RegionSummary%26region=2%26dataset=ABS_REGIONAL_ASGS2016%26geoconcept=ASGS_2016%26measure=MEASURE%26datasetASGS=ABS_REGIONAL_ASGS2016%26datasetLGA=ABS_REGIONAL_LGA2017%26regionLGA=LGA_2017%26regionASGS=ASGS_2016
 Neonatal intensive care is provided within a small number of specialized centers in metropolitan Melbourne. Abortion law differs between states in Australia. In Victoria, under the Abortion Law Reform Act 2008, abortion is permitted at the request of a woman up to 24 weeks. Past 24 weeks, two medical practitioners must agree that “abortion is appropriate in all the circumstances.”23Costa, C., Douglas, H., Hamblin, J., Ramsay, P., & Shircore, M. (2015). Abortion law across Australia – A review of nine jurisdictions. *Australian and New Zealand Journal of Obstetrics and Gynaecology, 55*(2), 105–111. https://doi.org/10.1111/ajo.12298. Participants in the four Victorian specialist children’s/maternity hospitals with tertiary or quaternary neonatal intensive care units were contacted by email during June–July 2018. We identified leading neonatologists/obstetricians in each institution who forwarded the invitation to colleagues regularly providing care to EPIs or their mothers. Non‐responders were sent two reminder emails. The questionnaire was anonymous. In return for completing the survey, participants had the opportunity to enter a draw to win a book voucher.

#### 2.1.2 Design

The survey was conducted using the online platform, Qualtrics. It consisted of four main sections (Appendix A: Figure A1). Section A explained the purpose and structure of the survey (including details of ethics approval) and asked potential participants to confirm their status as medical specialists and consent to the use of their data.

**Figure 1:**
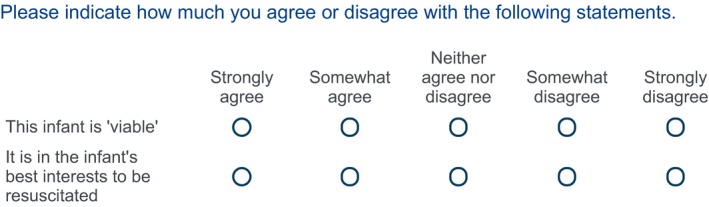
Example of survey question

Section B invited participants to reflect on current techniques for the treatment of women in extremely premature labor and any resulting infants. According to whether participants identified as specialists in obstetrics or neonatology, they were given questions with slightly different wording. For example, “Would you support Caesarean section” (for neonatologists) versus “Would you offer Caesarean” (for obstetricians). The responses were analyzed together. The initial questions related to a scenario with a woman in preterm labor at a non‐specified gestation (the fetus was otherwise normal) and participants were asked about the lowest or highest gestation that they would be willing to provide or support Caesarean section, abortion, resuscitation or non‐resuscitation (if the infant was born in a fair condition). They were then presented with scenarios (in random order) involving women in premature labor at gestations 22+3, 23+3 and 24+3 weeks. For each gestation, they were asked to estimate the chance of survival if resuscitation was attempted, and select their level of agreement (on a Likert scale from strongly agree to strongly disagree—see Figure 1) with statements that the infant was viable, that it was in its best interests to be resuscitated, and that they would support termination of pregnancy, resuscitation or non‐resuscitation at parental request.

Participants were asked about the definition of viability. They were asked separately about how each of the following elements were relevant to their understanding of “viability”: the proportion of surviving infants, dependence on technological support for survival (including whether or not technology needed to be available to the treating team for an infant to be regarded as viable), and the presence/absence of disability. Respondents were then asked to indicate their level of agreement with statements about recent developments in neonatal intensive care and how these advances impact on viability and the provision of various medical interventions.

**Figure 2:**
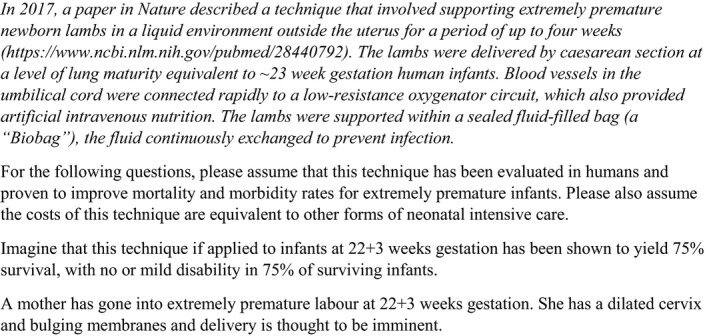
Ectogestation scenario

Section C introduced the concept of ectogestation by briefly describing the science behind this technique and invited participants to consider a hypothetical scenario where this technology had been shown to yield 75% survival with no or mild disability in 75% of surviving infants when applied at 22+3 weeks (Figure 2). (This is approximately equivalent to the outcome expected at 26 weeks gestation with current techniques.24Patel, R. M., Kandefer, S., Walsh, M. C., Bell, E. F., Carlo, W. A., Laptook, A. R., … Human Development Neonatal Research Network. (2015). Causes and timing of death in extremely premature infants from 2000 through 2011. *New England Journal of Medicine, 372*(4), 331–340. https://doi.org/10.1056/NEJMoa1403489.) They were then asked to select their degree of agreement with statements regarding management if this technology were available for an infant being delivered at 22+3 weeks gestation.

The final voluntary section included questions about participants’ basic demographics, professional experience, religiosity and general position towards abortion.

All participants who consented to the survey and answered at least one question were included in the analysis for the questions that they responded to, regardless of whether they completed the whole survey. Statistical analysis was carried out using RStudio Versions 3.5.1 and 3.5.325R Core Team. (2018). R: A language and environment for statistical computing. Vienna, Austria: R Foundation for Statistical Computing. Retrieved from https://www.R-project.org/
. The findings were descriptively presented as frequency (% of respondents for each question) for discrete variables and mean (standard deviation) for continuous variables. We generated graphs and tables to summarize these descriptive results. Graphs were generated using the package “ggplot2.”26Wickham, H. (2016). *Ggplot2: Elegant graphics for data analysis (Use R!)* (2nd ed.). Springer International. https://www.springer.com/gp/book/9783319242750



The association between medical specialty (obstetrics versus neonatology) for the Likert‐scale questions were examined using chi‐square tests for the difference between two proportions, by comparing those who agreed or disagreed, merging the “Strongly” and “Somewhat” categories, and discarding the “Neither agree nor disagree” responses. Chi‐square tests were also used to compare the proportion of obstetricians and neonatologists who selected particular gestations in Section B, compared to those who selected any other gestation including free‐text responses. We considered ordinal logistic regressions as an alternative to chi‐square tests to eliminate the need to merge categories. However, we ultimately decided against this strategy due to the sparsity of the data making the proportional odds assumption difficult to check. To compare the responses based on medical specialty for continuous variables, the *t* test was used. We elected not to examine for possible relationships between demographic characteristics and survey responses due to the small numbers in the study.

This project was approved by Human Research Ethics Committees (HREC) at Monash University, and the Mercy Hospital for Women, and the University of Oxford Central University Research Ethics Council.27Reference numbers: Monash University—12542; University of Oxford—R57714/RE001; Mercy Hospital—2018‐028. The other relevant health services (Royal Women’s Hospital, Royal Children’s Hospital and Monash Health) confirmed that additional site HREC approval was not required.

### 2.2 Results

#### 2.2.1 Sample

We contacted 143 professionals for the survey. There were 91 respondents who answered at least one question of the survey: 50 neonatologists and 41 obstetricians (response rates: 63% and 65%, respectively). Most respondents were between 30 and 60 years old. Two‐thirds were female. Sixty‐five percent were consultants, and 59% had at least 7 years of experience working with extremely premature infants (Table 1).

**Table 1:** Demographic characteristics of respondents

Characteristic	Response	Result *n* (%^a^)		
Professional specialty (*n* = 91)	Obstetrics	43 (46)		
	Neonatal	50 (54)		
		**Obstetrics**	**Neonatal**	**Total**
Age (*n* = 79)	18–30	5 (15)	2 (4)	7 (9)
	30–40	11 (32)	22 (49)	33 (42)
	40–50	10 (29)	14 (31)	24 (30)
	50–60	7 (21)	6 (13)	13 (17)
	60–70	–	1 (2)	1 (1)
	70+	1 (3)	–	1 (1)
Gender (*n* = 80)	Male	9 (26)	19 (41)	28 (35)
	Female	25 (74)	27 (59)	52 (65)
Professional role (*n* = 81)	Consultant	26 (74)	27 (59)	53 (65)
	Fellow	9 (26)	19 (41)	28 (35)
Years’ experience working with extremely premature infants (*n* = 81)	0–3	3 (8)	5 (11)	8 (10)
	3–7	11 (31)	14 (31)	25 (31)
	7–15	11 (31)	12 (27)	23 (28)
	15+	11 (31)	14 (31)	25 (31)
Self‐identification as belonging to a religion (*n* = 81)	Yes	12 (32)	18 (41)	30 (37)
	No (atheist)	15 (41)	18 (41)	33 (41)
	Not sure (agnostic)	10 (27)	8 (18)	18 (22)
Religion (*n* = 30)	Christianity	11 (92)	10 (56)	21 (70)
	Islam	–	6 (33)	6 (20)
	Judaism	1 (8)	–	1 (3)
	Buddhism	–	1 (6)	1 (3)
	Hinduism	–	1 (6)	1 (3)
	Other	–	–	–
Frequency religious services are attended (*n* = 29)	Never	3 (25)	6 (35)	9 (31)
	Once a month or less	6 (50)	3 (18)	9 (31)
	Twice a month or more	3 (25)	8 (47)	11 (38)
Importance of religion in respondents’ lives (*n* = 30)	Not at all important	1 (8)	1 (6)	2 (7)
	Slightly important	3 (25)	4 (22)	7 (23)
	Moderately important	3 (25)	5 (28)	8 (27)
	Very important	2 (17)	6 (33)	8 (27)
	Extremely important	3 (25)	2 (11)	5 (17)
General position towards abortion (*n* = 83)	Strongly pro‐life	–	2 (4)	2 (2)
	Moderately pro‐life	4 (11)	4 (9)	8 (10)
	Undecided	1 (3)	1 (2)	2 (2)
	Moderately pro‐choice	11 (30)	19 (41)	30 (36)
	Strongly pro‐choice	21 (57)	20 (43)	41 (49)

Note

Note that aside from professional specialty, answering demographic questions was optional.

a Not all percentages add to 100% due to rounding.

Thirty‐seven percent of respondents identified as being religious; 70% of these identifying as Christian. Religious respondents varied in the importance of religion to their lives (Table 1).

Most participants were moderately (36%) or strongly (49%) pro‐choice, whilst some identified as strongly pro‐life (2%), moderately pro‐life (10%) or undecided (2%). There was no significant difference between obstetric and neonatal respondents in their demographic characteristics, religion or views on abortion.

#### 2.2.2 Viability

Most surveyed doctors related the concept of viability to it being possible for an infant to survive at a given gestation (67%), indicated that the presence of disability was not relevant (78%) and included survival with medical intervention (100%) (Table 2). However, there was division relating to the accessibility of interventions: 49% thought that for a fetus/newborn to be viable the interventions had to be accessible to the infant and treating team, while 48% indicated that interventions need not be currently accessible.

**Table 2:** Respondents’ selections of conceptual elements of a definition of “viability”

Concept	Whether or not a fetus or newborn is considered viable at a particular gestation depends on…	Number of respondents^a^ (%)
The proportion of infants who survive	It is possible for infants to survive if born at this gestation	57 (67)
	The majority (>50%) of infants born at this gestation will survive	24 (28)
	The vast majority (>80%) of infants born at this gestation will survive	2 (2)
	Other	2 (2)
Survival with or without disability	Without disability	1 (1)
	Without severe disability	17 (20)
	With or without disability	66 (78)
	Other	1 (1)
Survival with or without medical intervention	Without medical intervention	0 (0)
	With medical interventions that are currently accessible to the infant and the treating team	42 (49)
	With medical interventions that could keep the fetus alive, even if they are not accessible to the infant	41 (48)
	Other	2 (2)

a Total number of respondents who answered each question = 85; not all percentages add to 100% due to rounding.

Participants were asked for their agreement with statements about viability and developments in medical practice. Seventy‐six percent of doctors agreed that the gestation at which an infant is considered viable had changed in the last 10 years. Most agreed that improvements in neonatal intensive care in the last decade changed how they felt about resuscitation of 23‐week infants (63%), although obstetricians were more likely to disagree than neonatologists (46% vs. 13%; χ^2^ = 10.7, *p* < .01; Appendix A: Figure A2). A minority of respondents (25%) indicated that these advances changed how they felt about abortion being offered to infants of the same gestation. The majority (53%) disagreed with the statement that laws on abortion should change as a consequence of changes in the viability of EPIs, while 24% neither agreed nor disagreed, and 24% agreed.

#### 2.2.3 Current practice

Most respondents (68%) selected 23 weeks as the lowest gestation at which they would resuscitate. Neonatologists were more willing than obstetricians to offer resuscitation at 22 weeks (20% vs. 5%; χ^2^ = 4.5, *p* = .03). The highest gestation at which most doctors would withhold resuscitation at parental request was between 24 and 25 weeks, inclusive (selected by 71% of obstetricians and 90% of neonatologists). However, some doctors were willing to withhold resuscitation at ≥26 weeks (4% of neonatologists and 22% of obstetricians; χ^2^ = 6.6, *p* < .01).

Most participants identified 23 or 24 weeks as the lowest gestation they would support Caesarean section for fetal reasons; however, neonatologists preferred lower gestations (71% selecting 22 or 23 weeks compared to 30% of obstetricians χ^2^ = 15.2, *p* < .01).

Obstetricians varied in the highest gestation that they would offer abortion at parental request when preterm birth is imminent. Although 47% of those who selected a gestation chose 24 weeks, eight chose “other.”

Estimated survival chances for infants born at 22+3, 23+3 and 24+3 weeks are presented in Appendix B: Table B1. For each gestation, the mean estimate was higher for neonatologists than obstetricians. For 22+3‐week infants, responses ranged from 0% to 75%.

**Figure 3:**
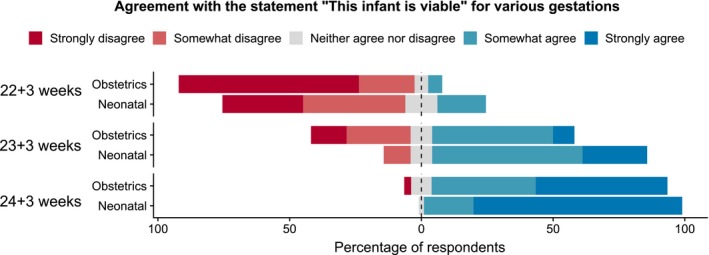
Agreement with the statement “This infant is viable” for hypothetical cases of preterm labor at different gestations. Obstetricians compared to neonatologists, for 22+3 weeks, χ^2^ = 3.8, *p* = .05; for 23+3 weeks χ^2^ = 9.6, *p* < .01 [Colour figure can be viewed at https://www.wileyonlinelibrary.com]

A large proportion (94%) of respondents agreed that a 24+3‐week infant was viable (Figure 3). There was less agreement at 23+3 weeks. Sixty‐nine percent of neonatologists and 89% of obstetricians did not believe that a 22+3‐week infant was viable. Overall, obstetricians were less likely than neonatologists to indicate that the hypothetical newborns were viable.

In the scenario, most surveyed doctors would be accepting of non‐resuscitation at 22+3, 23+3 or 24+3 weeks, although less so with increasing gestation (98%, 95%, 71%, respectively).

**Figure 4:**
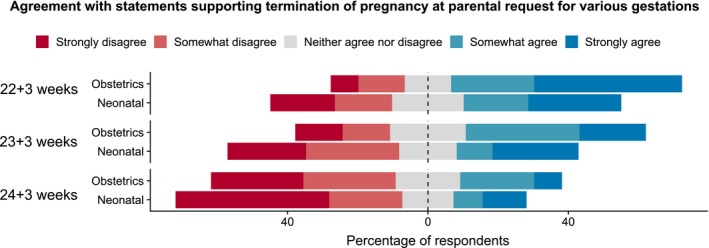
Agreement with statements supporting termination of pregnancy at parental request for various gestations [Colour figure can be viewed at https://www.wileyonlinelibrary.com]

There was little consensus when it came to supporting termination of pregnancy: for each gestation presented, responses ranged from strongly agree to strongly disagree (Figure 4). Overall, participants showed decreasing support of termination with increasing gestation.

#### 2.2.4 Ectogestation

**Figure 5:**
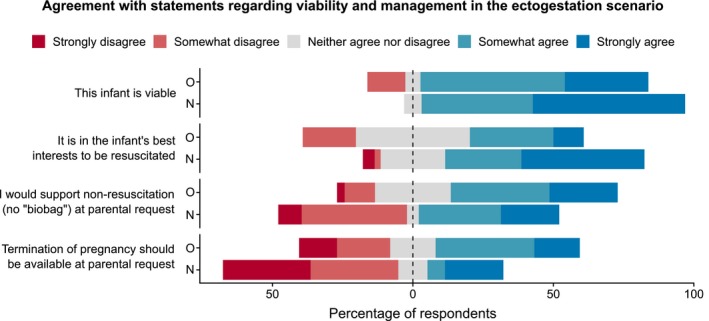
Agreement with statements regarding viability and management in the ectogestation scenario. O = obstetrics; N = neonatology [Colour figure can be viewed at https://www.wileyonlinelibrary.com]

Eighty‐eight percent of doctors agreed that in a hypothetical example of preterm labor and possible ectogestation at 22 weeks, they regarded the infant as “viable.” See Figure 5 whether doctors supported not using ectogestation if parents did not wish to use this technology was a point of contention, with 54% agreeing that they would support non‐provision of life‐prolonging treatment, 32% disagreeing and the remaining 14% neutral. Forty‐nine percent disagreed with abortion being an option in this scenario, and 13% neither agreed nor disagreed. Neonatologists were more likely to disagree than obstetricians (63% vs. 32%; χ^2^ = 7.1, *p* < .01).

Most respondents (61%) indicated that Caesarean in this scenario should be optional at 22 weeks; there was clear disagreement (93%) with a statement suggesting that this practice should become mandatory (Appendix A: Figure A3).

Participants were shown several statements regarding their general attitude towards ectogestation (Appendix A: Figure A4). Over half neither agreed nor disagreed that this technology should become common practice (55%), but 33% thought it should. Overall 41% of respondents agreed that this technology would influence their views on abortion being performed at 22 weeks, while 48% disagreed. There was uncertainty as to whether abortion law should change if ectogestation were to lower the age of viability; however, obstetricians were more likely to disagree with this idea compared to neonatologists (54% vs. 31%; χ^2^ = 4.4, *p* = .04).

### 2.3 Discussion of empirical findings

This survey asked Victorian obstetrics and neonatology specialists to consider questions relating to medical decision‐making at the borderline of viability. It is the first empirical study of neonatologists’/obstetricians’ conceptual understanding of viability. It is also the first survey of professionals’ views of ectogestation, and its potential impact on medical practice.

Key findings of the survey were that: doctors appeared to define viability differently to how they applied the concept; respondents indicated a belief that ectogestation would shift the gestational age of viability; they were divided as to whether ectogestation should become common practice; and they were unsure whether this technology should result in restrictions in access to abortion. The survey was limited by its small sample size (*n* = 91) and geographic setting (all respondents being from Victorian hospitals). Our study asked doctors’ views but did not provide an opportunity for participants to explain their reasoning (for example, we did not ask their views on consistency or the moral status of the fetus). Qualitative research would be helpful for providing further insight into doctors’ views on ectogestation, and the decision‐making process at the borderline of viability.

#### 2.3.1 Viability

When asked specifically about the criteria for the concept of viability, doctors appeared to support a view that the gestational age of viability should reflect the possibility of survival, regardless of disability. This contrasted with how the doctors applied the concept of viability. The clear majority did not believe that a 22+3‐week infant is viable (although they acknowledged that survival is possible at this gestation). Even at 23+3 weeks, a large proportion of obstetricians strongly or somewhat disagreed that a newborn was viable. This contrasts with the views of New York obstetricians and neonatologist in a 2008 survey, 90% of whom considered infants born ≥23 weeks gestation as viable,28Ramsay & Santella, op. cit. note 18. but aligns more closely with the results of a 2013 Canadian study, where 67% of participants identified 24 weeks to be the gestation of fetal viability.29Hull et al., op. cit. note 19.


Why do professionals apply the concept of viability in a way that contradicts their understanding of the concept? This could be the case for several reasons. Doctors may be unaware of the latest evidence regarding the survival of extremely premature infants, or their personal experience may not align with that evidence.

Doctors tended to agree with a statement suggesting that technology has lowered the gestational age of viability in the last 10 years; however, there was no consensus when it came to how this should influence medical practice. Most professionals indicated that improvements in survival did not change how they felt about termination of pregnancy being offered at 23 weeks, a finding that aligned with a previous survey of European obstetricians.30Habiba et al., op. cit. note 20. In our study, most obstetricians (but not most neonatologists) disagreed with a statement suggesting that a change in abortion law was required in response to improved survival for extremely preterm infants.

#### 2.3.2 Ectogestation

**Figure 6:**
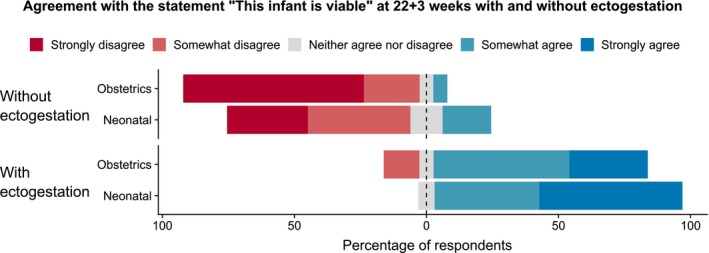
Agreement with the statement “This infant is viable” at 22+3 weeks with and without ectogestation [Colour figure can be viewed at https://www.wileyonlinelibrary.com]

Doctors who we surveyed agreed that ectogestation (in a hypothetical case example) would change the point of viability (Figure 6). However, when asked if ectogestation should be used to extend viability, or whether it should become common practice, doctors were ambivalent. The reasons for this hesitation were not clear.

Both obstetricians and neonatologists reported that the use of ectogestation in the scenario should not become mandatory, particularly when it was highlighted that this technology involves Caesarean section. The professionals had mixed views when it came to the permissibility of abortion (if ectogestation were available); there were doctors who strongly agreed and others who strongly disagreed with permitting abortion at the same gestation. There was a similar spread when doctors were asked whether ectogestation would influence laws and their personal feelings about abortion.

#### 2.3.3 Termination of pregnancy

The question of whether to offer termination of pregnancy to a woman in premature labor at her request proved controversial. When presented with the 22+3‐, 23+3‐ and 24+3‐week scenarios, for each case some obstetricians and neonatologists strongly agreed with termination of pregnancy, whilst others strongly disagreed. There was also a range of responses relating to how the concept of viability maps onto abortion, and whether viability should have any influence on this practice. In comparison, the majority of physicians in the Canadian study would “rarely” or “never” offer post‐viability termination of pregnancy in the absence of a “lethal” fetal abnormality.31Hull et al., op. cit. note 19.


In our study, obstetricians were more likely to support termination of pregnancy than neonatologists. Differences in attitudes between obstetricians/neonatologists may reflect different professional experience, as well as differences in perceived professional role: an obstetrician has a primary responsibility to the woman, a neonatologist to the infant.

## 3 ETHICAL ANALYSIS

The above results provide some insights into the way that practicing obstetricians and neonatologists understand and apply the concept of viability in the light of medical advances. However, these results, while valuable, do not settle the ethical question of how viability *should* be applied to neonatal and obstetric decisions. We will first, briefly, analyze the concept of viability. We will then examine the ethical implications of changes in viability for medical practice, drawing on one key ethical argument used in debates about viability.

### 3.1 Viability

It is not always clear what exactly is meant by viability. We have noted the intuitive idea that it reflects the point at which a fetus/newborn can survive outside the uterus. However, is it the point beyond which survival of newborns is possible, or likely? Does it depend on whether technology or techniques required to save infants are available? Is it just a question of survival, or do infants need to survive without a certain level of disability? For example, a newborn with the severe central nervous system abnormality anencephaly can be born with a heartbeat and will sometimes breathe for a short period after birth. However, such infants do not usually survive for long.32With provision of intensive care, survival for more than 1 year has been described. Wilkinson, D. J., Thiele, P., Watkins, A., & De Crespigny, L. (2012). Fatally flawed? A review and ethical analysis of lethal congenital malformations. *BJOG: An International Journal of Obstetrics & Gynaecology, 119*(11), 1302–1308. https://doi.org/10.1111/j.1471-0528.2012.03450.x. Is an anencephalic fetus viable?

Definitions of viability in published literature vary considerably (Appendix B: Table B2). Table 3 identifies and labels a range of possible definitions that might be applied, with gestations ranging from 21 to 32 weeks. Clearly, if viability is taken to have ethical significance it would be important to be clear about which definition should be used.

**Table 3:** Possible definitions of viability and approximate corresponding gestational ages

Concept	**Definition**	**Gestational age**
Absolute Viability (AV)	The youngest gestation with a known survivor with or without disability anywhere in the world	21 weeks and 4 days^a^
Median Viability (MV)	The gestation at which >50% of infants will survive with or without disability with the medical intervention available to them	Somewhere between 23 and 25 weeks in a neonatal intensive care unit in a developed country^b^
Median Intact Viability (MIV)	The gestation at which >50% of infants will survive, and those infants will be free from major disability with the medical intervention available to them	Around 26 weeks^c^, although depends on definition of major disability (and also varies with intensity of interventions provided and how often treatment is withdrawn because of predicted disability)
Natural Viability (NV)	The gestation at which >50% of infants will survive (with or without disability) in the absence of major medical intervention (e.g., mechanical ventilation and intensive care)	This definition is hard to apply to high‐income countries as after a certain gestation it would be regarded as unethical to not provide intervention. From countries without neonatal intensive care it could be estimated to be around 34 weeks^d^

a Ahmad, K. A., Frey, C. S., Fierro, M. A., Kenton, A. B., & Placencia, F. X. (2017). Two‐year neurodevelopmental outcome of an infant born at 21 weeks’ 4 days’ gestation. *Pediatrics, 140*(6), e20170103. https://doi.org/10.1542/peds.2017-0103.

b Patel, op. cit. note 6.

c Ibid.

d Platt, M. (2014). Outcomes in preterm infants. *Public Health, 128*(5), 399–403.

In neonatology, there is not a single threshold of viability. Instead, there are at least two different thresholds—a level of maturity beyond which prognosis is sufficiently high that resuscitation and provision of life‐prolonging treatment is mandatory (sometimes referred to as the “Upper Threshold”),33Wilkinson, D., Verhagen, E., & Johansson, S. (2018). Thresholds for resuscitation of extremely preterm infants in the UK, Sweden, and Netherlands. *Pediatrics, 142*(Suppl 1), S574–S584. https://doi.org/10.1542/peds.2018-0478I. and a level of maturity below which the prognosis is sufficiently poor that resuscitation is not an option (the “Lower Threshold”). In between these two thresholds, resuscitation and intensive care provision is seen to be optional. The Lower Threshold in developed countries is usually regarded as being at 22–24 weeks (varying between countries).34Ibid. It is thus close to, but not coinciding with Absolute Viability (Table 3). The Upper Threshold for resuscitation is understood to lie at 23–26 weeks.35Ibid. It appears to correspond with something like Median Viability or Median Intact Viability.36One possible reason for a difference between countries in where they set the Upper Threshold for neonatal resuscitation could be the use of MIV rather than MV as the basis (ibid).


In obstetrics, viability is invoked in some countries’ abortion law as both a hard limit after which abortion is not legal (notably in the United States37Han et al., op. cit. note 11.), and implicitly as the underpinning of a limit, without being expressly referred to. In some jurisdictions, viability is not explicitly stated in the law; however, gestational restrictions may exist at the policy or implementation level.38Erdman, op. cit. note 9. For example, many jurisdictions (e.g. UK, Victoria [Australia]) use 24 weeks as a cut‐off for abortion.39Han et al., op. cit. note 11. In both Victoria and the UK, abortion is permitted after 24 weeks; however, a different standard or process is required for access beyond this gestation.


Which definition of viability is used to determine the permissibility of abortion may depend on the ethical basis for drawing such a line. There are two principal arguments in favor of limiting or prohibiting abortion beyond the point of viability. Some believe that viability confers *moral status*, and therefore post‐viability abortions are the equivalent of infanticide.40Jensen, op. cit. note 9; Chervenak et al., op. cit. note 13; McMahan, J. (2013). Infanticide and moral consistency. *Journal of Medical Ethics, 39*(5), 273–280. Others maintain that doctors should be *consistent* in the way that they treat a fetus and newborn infant of the same level of maturity: once a pregnancy is sufficiently advanced that doctors would resuscitate premature newborns, abortion should no longer be permitted.41Gillon, R. (2001). Is there a ‘new ethics of abortion’? *Journal of Medical Ethics, 27*(Suppl 2), ii5.


It is worth noting first that many on both sides of the abortion debate reject viability as significant for the permissibility of abortion. Those with strongly pro‐life views argue that an early embryo has full moral status and therefore would disallow termination of pregnancy even before the point of viability; those who are strongly pro‐choice often believe that the ethical significance of a woman’s autonomy means that termination should be an option beyond viability, or may argue that even the late‐term fetus lacks moral status. On any of these views, technologies that alter the point of viability (such as ectogestation or more conventional medical advances) should make no ethical difference to policy or law around abortion.42As noted in Section 1, a number of countries’ (e.g., Finland, Belgium, Denmark) abortion policies use cut‐off points prior to viability. It is beyond the scope of this paper to discuss the ethical basis for such policies; however, changes in viability would not be expected to influence policy in those countries.


For the sake of this paper, we will set aside arguments that viability confers moral status,43It is beyond the scope of this paper, but we are sceptical of such arguments. Moral status confers a stringent protection against being killed. If the fetus has moral status it would be wrong to kill it, (absent very special circumstances). However, it is not clear how viability could be important for this sort of moral consideration. Viability refers to the ability of a newborn to survive outside of the womb (usually with considerable medical and technological support). However, the relevant question for abortion is survival within the womb, not outside the womb. In the vast majority of situations where abortion is sought, survival of the fetus would be possible by continuing the pregnancy. Many adults are not “viable,” in the sense that they are dependent on medical technology for survival (ventilators, dialysis machines, or just the provision of medicines like insulin). However, we do not ordinarily think that those who are dependent on medical technology or on others for their survival lose their protection against being killed. to focus on the argument from consistency.44Fost et al., op. cit. note 9, p. 6; McMahan, op. cit. note 40. Briefly, the intuitive argument is that it appears inconsistent to allow some doctors to kill fetuses that have reached a level of maturity where, if born alive, other doctors would be ethically obliged to resuscitate them. On one view, such inconsistency should be avoided by aligning abortion policy with neonatal care.45Although, we have separated these two arguments, they clearly overlap to some degree. A desire for consistency may arise from a recognition of moral status. (We are grateful to a reviewer for highlighting this.) We will not discuss them in detail here, but there are also problems with the consistency argument. For example, the ethically relevant difference between the fetus in the uterus and the newborn ex‐utero, is that in the latter but not the former case treatment and medical care can be provided without infringing or compromising the autonomy of the woman. This ethical difference means that it is not necessarily inconsistent to treat the fetus and newborn differently at the same point in pregnancy.


If consistency is the ethical basis for abortion policy, we can assess the implications of a technology like ectogestation by focusing first on its implications for newborn resuscitation and provision of intensive care. What would or should those implications be?

### 3.2 Ectogestation

As we noted above, there are two different normative thresholds for treatment of newborns—the Lower and Upper Thresholds. The Lower Threshold is the point beyond which resuscitation and intensive care is *permissible*. It reflects the judgement that treatment is potentially in the best interests of the newborn. As noted in Section 1, it appears possible that ectogestation could move the Lower Threshold downwards by making it possible for infants to survive who would previously have been too small and immature to do so.

Would ectogestation also lower the Upper Threshold? If the technology dramatically improved the outcome of EPIs, it might shift the points at which the majority of infants would survive (with or without impairment). Conceivably, Median Viability or Median Intact Viability might provide the normative basis for the Upper Threshold.46Wilkinson, D. (2016). The grey zone in neonatal treatment decisions. In R. McDougall, C. Delany, & L. Gillam (Eds.), When Doctors and Parents Disagree: Ethics, Paediatrics and the Zone of Parental Discretion (Chapter 4). Sydney, Australia: Federation Press. However, there is another important consideration. The ectogestation technique as described by the Philadelphia group requires delivery by Caesarean section (CS).47There are potentially several reasons for this. A significant proportion of extremely premature infants around 22/23 weeks gestation die during labor because they do not cope with the stress of delivery. Furthermore, the aim of the ectogestation technique is to maintain fetal circulation as it would normally be within the uterus (oxygenation and nutrient supply via the placenta rather than via lungs and gut). Birth triggers transition from fetal to neonatal circulation. Once birth has occurred it may not be possible to reverse those changes and restore a fetal circulation. That is significant because in most jurisdictions, women are not ethically obliged to undergo Caesarean, even if that would lead to the survival of a newborn. It would be regarded as an unacceptable breach of the woman’s autonomy to perform such a procedure against her wishes (assuming she has capacity);48American College of Obstetricians & Gynecologists’ Committee on Ethics. (2016). Committee Opinion No. 664: Refusal of medically recommended treatment during pregnancy. *Obstetrics and Gynecology, 127*(6), e175. accordingly, it appears that ectogestation should be regarded as ethically optional.49It is unclear at this point what the risks to a woman would be of delivering by cesarean section in the setting of ectogestation. However, Caesareans in mid‐gestation (before development of the lower uterine segment) is associated with greater risks of serious complications such as bleeding and uterine rupture in subsequent pregnancies. Evans, L. C., & Combs, C. A. (1993). Increased maternal morbidity after cesarean delivery before 28 weeks of gestation. *International Journal of Gynaecology & Obstetrics, 40*(3), 227–233; Subramanian, R., Mishra, P., Subramaniam, R., & Bansal, S. (2018). Role of anesthesiologist in ex utero intrapartum treatment procedure: A case and review of anesthetic management. *Journal of Anaesthesiology Clinical Pharmacology*, 34(2), 148–154. If a mother did not wish to deliver by Caesarean, only standard intensive care would be possible for the newborn.

What would this mean, then, for obstetric care and abortion?

If viability cut‐offs for abortion are justified by a desire for consistent treatment of the fetus and newborn, then the relevant viability threshold for abortion policy should be aligned with the neonatal *Upper Threshold.* Before this point, it would be permissible for a woman in premature labor to request that doctors do not resuscitate her EPI, accepting that would lead to the death of the newborn. It would appear ethically consistent to also allow the woman to choose to discontinue her pregnancy and seek an abortion.50These two decisions are not completely symmetric. Those who regard it as more problematic to end the life of a fetus than to refrain from resuscitating a newborn will not necessarily wish to align them in this way. However, those who hold such a view are also likely to reject the significance of viability at all. Cohen, op. cit. note 8. Beyond the Upper Threshold, newborn resuscitation and intensive care becomes mandatory (and will be provided even if parents do not wish it). Accordingly, it could be potentially ethically consistent to also prohibit abortion beyond this point.51Those who reject the consistency argument will not necessarily accept this. Fost et al. ask: “Why should a fetus's capacity to live independently be a reason to forbid the mother from forcing it to live independently?”. Fost et al., op. cit. note 9.


We have argued that ectogestation, if successfully translated into human neonatal care might affect the Lower Threshold, but not the Upper Threshold for neonatal resuscitation/provision of intensive care; it would increase the permissibility of treatment at extremely low gestational ages, but not make such treatment mandatory. It follows, that on the above consistency argument, ectogestation should *not* change the relevant viability threshold for abortion policy. In Victoria, Australia (and in other places like the UK), the legislative cut‐off for permitting/restricting abortion in law is currently 24 weeks gestation. If the relevant justification for policy is a desire for consistency, it appears that there would be no ethical reason to revise policy based on ectogestation becoming available.

It is important to note that some, but not all, of the above conclusions are relevant to improvements in outcome for EPIs that arise from more conventional advances in neonatal intensive care. Improvements in outcome over the last two decades have made it possible for EPIs to survive earlier than had been possible. They appear to have led to a shift in the Lower Threshold for resuscitation.52Wilkinson et al., op. cit. note 33. What of the Upper Threshold? Unlike ectogestation, standard neonatal intensive care does not require a woman to undergo Caesarean section. Therefore, the argument arising from the right to refuse surgical intervention does not apply. It may be thought that advances in standard neonatal intensive care would have led to changes in the neonatal Upper Threshold, and, (if the consistency argument is thought relevant) would warrant some modification of abortion policy. However, women have a right to refuse other obstetric interventions that could significantly contribute to improved neonatal outcome.53While women in premature labour at, say, 24 weeks gestation do not usually require Caesarean section, there are other interventions that are usually given that significantly improve the outcome for the newborn. For example, women are usually transferred to deliver in specialized tertiary centres with neonatal intensive care facilities. They are given intramuscular injections of steroids. If a woman in preterm labour at 24 weeks gestation decided to decline steroids and elected to deliver at home or in a birthing centre, it may then be that the outcome for the newborn would be sufficiently poor that palliation would be permissible. As a consequence, palliative care and non‐resuscitation may still be an option for infants of 24 or even 25 weeks gestation; in practice, the Upper Threshold may not have changed in the last two decades.

### 3.3 Reflective equilibrium

It is potentially useful to re‐examine our survey responses in the light of the above analysis. We described four different theoretical definitions of viability. Our survey respondents understood viability in the abstract as reflecting the possibility of survival (Absolute Viability); however, when asked specifically whether an infant at a specific gestation was “viable” they appeared to use a higher threshold, (possibly reflecting Median Viability or Median Intact Viability). It may be that they were answering the latter question with the Upper Threshold (and abortion) in mind.

Our respondents appeared to anticipate that ectogestation would shift the Lower Threshold for neonatal resuscitation. In the hypothetical case scenario of an infant at 22+3 weeks gestation, the majority endorsed the provision of intensive care with ectogestation, if parents desired this, although few supported treatment at this gestation with current technology and outcomes. However, very few professionals indicated that Caesarean section for ectogestation should be mandatory, implicitly accepting that ectogestation itself would be ethically optional. Survey respondents were divided in their views on whether ectogestation should influence laws on abortion; however, a majority of obstetricians did not think that the law should change, which might be a reflection of support for the argument that we have developed above. Alternatively, it may reflect a view that access to abortion should not be linked to viability.

## 4 CONCLUSIONS

This paper sought to explore the ethical implications of ectogestation. We have focused on the significance of ectogestation for viability, since this technique could make it possible for extremely premature infants to survive outside the uterus who would previously have been unable to do so.

We have combined empirical and analytical approaches to examining the implications of ectogestation for viability, neonatal resuscitation and abortion. We surveyed practicing medical specialists in Victoria on ectogestation and medical decision‐making at the borderline of viability. Surveyed doctors appeared to apply the concept of viability in a way that was different from their theoretical understanding. Professionals believed that ectogestation would shift the gestational age of viability; however, they were divided as to whether this technology should become common practice and were unsure whether it should result in restrictions in access to abortion.

Our ethical analysis has clarified the concept of viability. We suggested that ectogestation would alter the Lower, but not the Upper Threshold for neonatal resuscitation and provision of life‐prolonging treatment, increasing the potential permissibility of resuscitation at extremely low gestational age, but not making it mandatory. For abortion, we concentrated on the argument from consistency. Our aim was not to defend this argument; nevertheless, if consistency is the ethical basis for abortion policy, our analysis suggests that ectogestation would not necessarily warrant changes in cut‐off gestations for abortion.

Moving forward, qualitative research would be useful to understand the reasons behind professionals’ views on viability, abortion and newborn care. This would assist in informing further ethical deliberation and reflective equilibrium on ectogestation and related advances in neonatal care.

## Data Availability

Data from this research project will be accessible after publication at the Oxford University Research Archive: ORA‐data.

