# Supplementary material for: Ectogestation ethics: The implications of artificially extending gestation for viability, newborn resuscitation and abortion
Source: Bioethics. 2019 Nov 7;34(4):371–84. doi: 10.1111/bioe.12682 (PMC7216952; doi:10.1111/bioe.12682)
Supplement: Supplementary file 1 [file BIOE-34-371-s001.docx]

**Appendices**

APPENDIX A: FIGURES


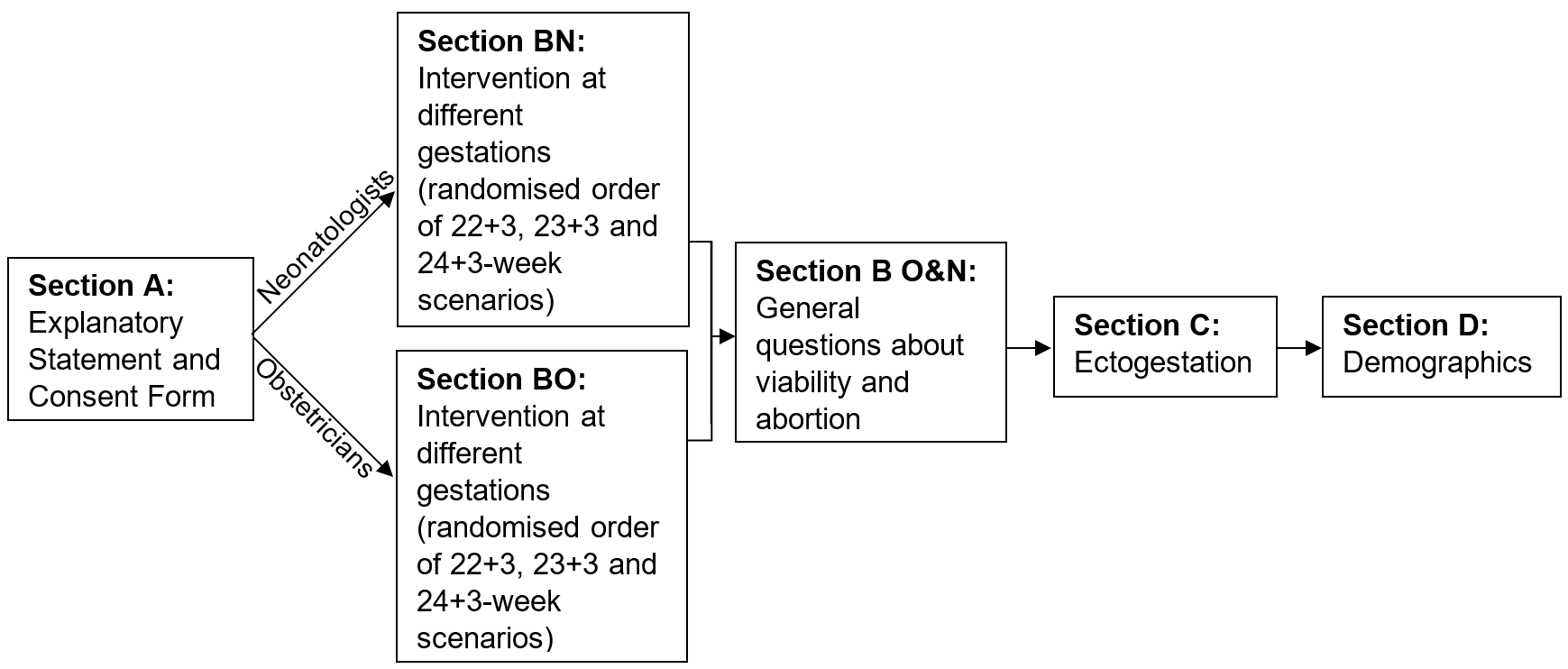
 **Figure A1** Survey flow


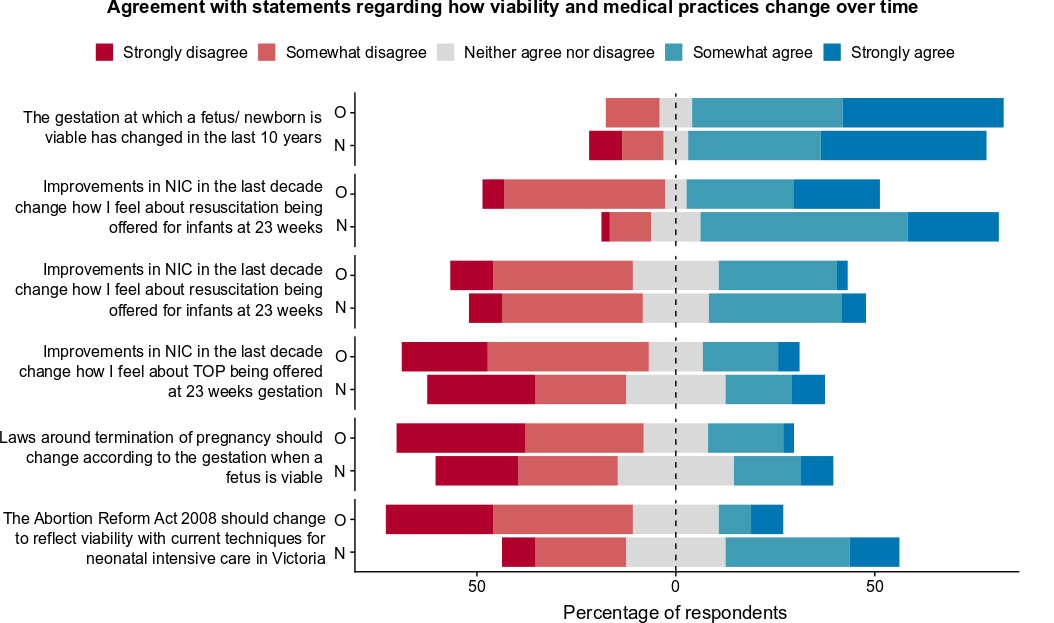
 **Figure A2** Agreement with statements regarding how viability and medical practices change over time. NIC = neonatal intensive care; TOP = termination of pregnancy; O = obstetrics; N = neonatology


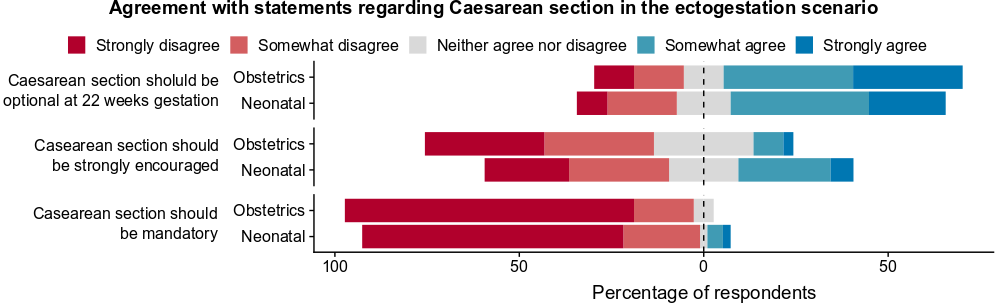
 **Figure A3.** Agreement with statements regarding ectogestation and Caesarean section


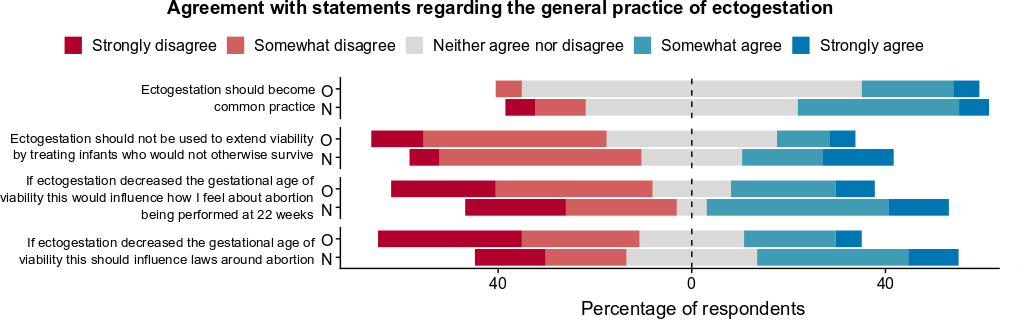
 **Figure A4.** Agreement with statements regarding the general practice of ectogestation. O = obstetrics; N = neonatology

APPENDIX B: TABLES

**Table B1** Estimated chance of survival at various gestations: mean, standard deviation and *t* test

| **Gestation** | **Obstetrics (O) or neonatology (N)** | **Mean chance of survival (%)** | **Standard deviation** | ***t* test**^a^ |
| --- | --- | --- | --- | --- |
|  |  |  |  |  |
| 22+3 weeks | O (*n* = 34) | 7 | 12.1 | *t* = 1.9  *p* = .06 |
|  | N (*n* = 41) | 13 | 14.6 |  |
|  |  |  |  |  |
|  |  |  |  |  |
| 23+3 weeks | O (*n* = 34) | 20 | 14.3 | *t* = 3.4  *p* = .001 |
|  | N (*n* = 49) | 32 | 15.7 |  |
|  |  |  |  |  |
|  |  |  |  |  |
| 24+3 weeks | O (*n* = 35) | 38 | 18.6 | *t* = 2.7  *p* = .01 |
|  | N (*n* = 47) | 48 | 15.0 |  |
|  |  |  |  |  |

^a^Unpaired *t* test with two-sided *p* value

**TABLE B2** Examples of attempts to define viability

| **Reference** | **Attempt to define viability** |
| --- | --- |
| Fost et al., 1980a | - “…viability is the capacity of an organism to survive for a designated period of time in a defined environment” |
| Gert, 1995b | - “…the age of viability being the earliest age at which any fetus has been known to survive outside its gestational mother's womb” - “A fetus is viable when it is reasonably likely that it will survive outside its mother's womb without suffering a serious handicap as a result of its removal” |
| Gillon, 2001c | - “…the concept of viability – the stage of fetal development at which the fetus can survive independently of the pregnant woman, given suitable intensive care” |
| Glass et al., 2015d | - “Human viability, defined as [the] gestational age at which the chance of survival is 50%, is currently approximately 23–24 weeks in developed countries” |
| Jensen, 2015^e^ | - “…whether a fetus has meaningful viability depends on whether an infant of the same age born prematurely in the same circumstances would ceteris paribus enjoy a reasonably healthy life” |
| Erdman, 2017f | - “…viability, defined as the point at which the fetus is capable of sustained life outside the uterus, with or without artificial aid” |
| Romanis, 2018g | - “...viability, a concept referring to the ability of a developing human being to survive ex utero” |

aFost, N., Chudwin, D., & Wikler, D. (1980). The limited moral significance of ‘fetal viability’. *Hastings Center Report, 10*(6), 10–13. doi:10.2307/3560289. bGert, H. J. (1995). Viability. *International Journal of Philosophical Studies, 3*(1), 133–142. doi:10.1080/09672559508570807. cGillon, R. (2001). Is there a ‘new ethics of abortion’? *Journal of Medical Ethics, 27*(Suppl 2), ii5. dGlass, H. C., Costarino, A. T., Stayer, S. A., Brett, C. M., Cladis, F., & Davis, P. J. (2015). Outcomes for extremely premature infants. *Anesth Analg, 120*(6), 1337–1351. eJensen, D. (2015). Birth, meaningful viability and abortion. *Journal of Medical Ethics, 41*(6), 460-463. fErdman, J. N. (2017). Theorizing time in abortion law and human rights. *Health and Human Rights, 19*(1), 29–40. gRomanis, E. C. (2018). Artificial womb technology and the frontiers of human reproduction: Conceptual differences and potential implications. *Journal of Medical Ethics, 44*(11), 751–755. doi:10.1136/medethics-2018-104910

APPENDIX C: SURVEY

**Section A: Explanatory Statement and Consent Form**

Oxford Uehiro Centre for Practical Ethics, Littlegate House, St Ebbes St, Oxford OX1 1PT, Tel 01865 286 888

**Study title:** Medical treatment at the borderline of viability, Victorian Ethics Survey 2018

**Purpose:** To pilot a survey which will be used to determine the views of specialist Victorian doctors (who work with extremely premature infants or their mothers) on ethical questions relating to medical treatment at the borderline of viability.

**Background:** Advances in medical care of extremely premature infants can lead to challenging ethical questions for professionals who care for pregnant women and critically ill newborns.

We are interested to understand the views of Victorian doctors who work in obstetrics or neonatal intensive care about these questions. We are asking neonatal and obstetric consultants at tertiary centres in Melbourne to take part and would like all of this group to have their say.

We would really value your perspective on these issues.

**Survey:** This is an online survey entitled Medical treatment at the borderline of viability, Victorian Ethics Survey 2018 that should take approximately 15-20 minutes. The survey asks you to consider scenarios relating to neonatal and obstetric management in the light of current and future technology. This includes questions about neonatal resuscitation as well as about termination of pregnancy.

This survey is anonymous and we cannot trace answers back to you or your computer. No identifying details or details of individual hospitals will be recorded.

The University of Oxford CUREC (R57714/RE001) If there are any issues with this The University of Oxford CUREC (R57714/RE001). If there are any issues with this survey please contact Prof Dominic Wilkinson at the Uehiro Centre for Practical Ethics (dominic.wilkinson@philosophy.ox.ac.uk).

To enter the draw to win a Readings gift voucher (value $200), please follow the link to enter your email address at end of the survey (this information will be stored separately to survey results to preserve anonymity).

Have you read the terms above, and agree to participate in the survey?

Yes

No

Display This Question:

If Have you read the terms above, and agree to participate in the survey? = No

You have indicated that you do not agree to participate in the survey. Was this an

error? By selecting 'No', you will be taken to the end of the survey.

Yes

No

Skip To: End of Survey If You have indicated that you do not agree to participate in the survey. Was this an error? By... = No

Display This Question:

If You have indicated that you do not agree to participate in the survey. Was this an error? By... = Yes

Have you read the terms above, and agree to participate in the survey?

Yes

No

Skip To: End of Survey If Have you read the terms above, and agree to participate in the survey? = No

Are you over the age of 18, and does your professional medical practice involve care of extremely premature infants or their mothers (ie consultant or registrar/fellow in obstetrics or neonatology)?

Yes

No

Display This Question:

If Are you over the age of 18, and does your professional medical practice involve care of extrem... = No

You have indicated that your professional medical practice does not involve care of extremely premature infants or their mothers. Was this an error?

By selecting 'No', you will be taken to the end of the survey.

Yes

No

Skip To: End of Survey If 2.2. You have indicated that your professional medical practice does not involve care of extremel... = No

Display This Question:

If You have indicated that your professional medical practice does not involve care of extremel... = Yes

Are you over the age of 18, and does your professional medical practice involve care of extremely premature infants or their mothers (ie consultant or registrar/fellow in obstetrics or neonatology)?

Yes

No

Skip To: End of Survey If Are you over the age of 18, and does your professional medical practice involve care of extrem... = No

# Specialty

What is your professional specialty?

Neonatal

Obstetrics

# Section B N 1

For the first part of the survey, we would like you to reflect on currently available techniques for treatment of premature infants and their implications.

For the following questions, please assume that an early dating scan has previously been performed to estimate gestational age, and that there are no other known medical conditions affecting the fetus or mother.

A mother has gone into extremely premature labour.

She has been counselled and received accurate information about the chance of survival if the infant is born in the next 24 hours, about potential complications of intensive care, and about the risk of long-term neurodevelopmental problems if the

infant survives.

Imagine that the infant is born in fair condition.

NB. Numbers below indicate completed weeks of gestation - i.e. X+0 weeks.

If the parents request active resuscitation, what is the lowest gestational age at which you would be prepared to attempt resuscitation (including intermittent positive pressure ventilation and intubation)? (i.e. if the infant is more premature than this you would not resuscitate).

Please move the circle to indicate gestation.

Not Applicable

20 21 22 23 24 25 26

Lowest gestational

age (weeks)

If other, please specify, e.g. X weeks and Y days.

If the parents request non-resuscitation, what is the highest gestational age at which you would be prepared to NOT attempt resuscitation (including intermittent positive pressure ventilation and intubation)? (i.e. if the infant is less premature than this you would resuscitate against parental wishes).

Not Applicable

20 21 22 23 24 25 26

Highest gestational

age (weeks)

If other, please specify, e.g. X weeks and Y days.

The parents are requesting all possible measures to improve the chance of survival for the infant and have received accurate information about the risks and benefits of treatment options.

What is the lowest gestational age at which you would be prepared to support caesarean section? (ie. If the fetus is more premature than this, caesarean section should not be provided even if requested)

Not Applicable

20 21 22 23 24 25 26

Lowest gestational

age (weeks)

If other, please specify, e.g. X weeks and Y days.

# Section B N 2 22+3

A mother has gone into extremely premature labour at **22+3 weeks** gestation. She has a dilated cervix and bulging membranes and delivery is thought to be imminent. She has received a full course of antenatal steroids.

She has been counselled and received accurate information about the chance of survival if the infant is born in the next 24 hours, about potential complications of intensive care, and about the risk of long-term neurodevelopmental problems if the infant survives.

If the infant is born and resuscitation is attempted, what is your estimate for the chance of survival without severe disability (to the nearest 5%)?

Not Applicable

0 5 10 15 20 25 30 35 40 45 50 55 60 65 70 75 80 85 90 95 100

Percentage chance

of survival without

severe disability

Please indicate how much you agree or disagree with the following statements.

|  | Strongly agree (1) | Somewhat agree (2) | Neither agree nor disagree (3) | Somewhat disagree (4) | Strongly disagree (5) |
| --- | --- | --- | --- | --- | --- |
| This infant is 'viable' |  |  |  |  |  |
| It is in the infant's best interests to be resuscitated |  |  |  |  |  |

Imagine that the parents are concerned about the risk of death despite treatment, or survival with severe disability. They are requesting termination of pregnancy.

Please indicate how much you agree or disagree with the following statement.

|  | Strongly agree (1) | Somewhat agree (2) | Neither agree nor disagree (3) | Somewhat disagree (4) | Strongly disagree (5) |
| --- | --- | --- | --- | --- | --- |
| This option should be available to them (1) |  |  |  |  |  |

# Section B N 2 23+3

A mother has gone into extremely premature labour at **23+3 weeks** gestation. She has a dilated cervix and bulging membranes and delivery is thought to be imminent. She has received a full course of antenatal steroids.

She has been counselled and received accurate information about the chance of survival if the infant is born in the next 24 hours, about potential complications of intensive care, and about the risk of long-term neurodevelopmental problems if the infant survives.

If the infant is born and resuscitation is attempted, what is your estimate for the chance of survival without severe disability (to the nearest 5%)?

She has been counselled and received accurate information about the chance of survival if the infant is born in the next 24 hours, about potential complications of intensive care, and about the risk of long-term neurodevelopmental problems if the infant survives.

If the infant is born and resuscitation is attempted, what is your estimate for the chance of survival without severe disability (to the nearest 5%)?

Not Applicable

0 5 10 15 20 25 30 35 40 45 50 55 60 65 70 75 80 85 90 95 100

Percentage chance

of survival without

severe disability

Please indicate how much you agree or disagree with the following statements.

|  | Strongly agree (1) | Somewhat agree (2) | Neither agree nor disagree (3) | Somewhat disagree (4) | Strongly disagree (5) |
| --- | --- | --- | --- | --- | --- |
| This infant is 'viable' |  |  |  |  |  |
| It is in the infant's best interests to be resuscitated |  |  |  |  |  |

Imagine that the parents are concerned about the risk of death despite treatment, or survival with severe disability. They are requesting termination of pregnancy.

Please indicate how much you agree or disagree with the following statement.

|  | Strongly agree (1) | Somewhat agree (2) | Neither agree nor disagree (3) | Somewhat disagree (4) | Strongly disagree (5) |
| --- | --- | --- | --- | --- | --- |
| This option should be available to them (1) |  |  |  |  |  |

# Section B N 2 24+3

A mother has gone into extremely premature labour at **24+3 weeks** gestation. She has a dilated cervix and bulging membranes and delivery is thought to be imminent. She has received a full course of antenatal steroids.

She has been counselled and received accurate information about the chance of survival if the infant is born in the next 24 hours, about potential complications of intensive care, and about the risk of long-term neurodevelopmental problems if the infant survives.

If the infant is born and resuscitation is attempted, what is your estimate for the chance of survival without severe disability (to the nearest 5%)?

She has been counselled and received accurate information about the chance of survival if the infant is born in the next 24 hours, about potential complications of intensive care, and about the risk of long-term neurodevelopmental problems if the infant survives.

If the infant is born and resuscitation is attempted, what is your estimate for the chance of survival without severe disability (to the nearest 5%)?

Not Applicable

0 5 10 15 20 25 30 35 40 45 50 55 60 65 70 75 80 85 90 95 100

Percentage chance

of survival without

severe disability

Please indicate how much you agree or disagree with the following statements.

|  | Strongly agree (1) | Somewhat agree (2) | Neither agree nor disagree (3) | Somewhat disagree (4) | Strongly disagree (5) |
| --- | --- | --- | --- | --- | --- |
| This infant is 'viable' |  |  |  |  |  |
| It is in the infant's best interests to be resuscitated |  |  |  |  |  |

Imagine that the parents are concerned about the risk of death despite treatment, or survival with severe disability. They are requesting termination of pregnancy.

Please indicate how much you agree or disagree with the following statement.

|  | Strongly agree (1) | Somewhat agree (2) | Neither agree nor disagree (3) | Somewhat disagree (4) | Strongly disagree (5) |
| --- | --- | --- | --- | --- | --- |
| This option should be available to them (1) |  |  |  |  |  |

# Section B O 1

For the first part of the survey, we would like you to reflect on currently available techniques for treatment of premature infants and their implications.

For the following questions, please assume that an early dating scan has previously been performed to estimate gestational age, and that there are no other known medical conditions affecting the fetus or mother.

A mother has gone into extremely premature labour.

She has been counselled and received accurate information about the chance of survival if the infant is born in the next 24 hours, about potential complications of intensive care, and about the risk of long-term neurodevelopmental problems if the

infant survives.

NB. Numbers below indicate completed weeks of gestation - i.e. X+0 weeks.

Imagine that the parents are concerned about the risk of death despite treatment, or survival with severe disability. They are requesting termination of pregnancy. What is the highest gestational age at which you would be prepared to provide termination of pregnancy? (i.e. if the infant is less premature than this you would not be prepared to

provide termination of pregnancy).

Please move the circle to indicate gestation.

Not Applicable

20 21 22 23 24 25 26

Lowest gestational

age (weeks)

If other, please specify, e.g. X weeks and Y days.

Imagine that the parents are requesting all possible measures to improve the chance of survival for the infant and have received accurate information about the risks and benefits of treatment options.

What is the lowest gestational age at which you would be prepared to perform caesarean section for fetal (non-maternal) reasons? (ie. If the fetus is more premature than this, caesarean section should not be provided even if requested)

Please move the circle to indicate gestation.

Not Applicable

20 21 22 23 24 25 26

Lowest gestational

age (weeks)

If other, please specify, e.g. X weeks and Y days.

If the parents request active resuscitation, what is the lowest gestational age at which you would support attempted resuscitation by a neonatologist (including intermittent positive pressure ventilation and intubation)? (i.e. if the infant is more premature than this you would not support active resuscitation).

Please move the circle to indicate gestation.

Not Applicable

20 21 22 23 24 25 26

Lowest gestational

age (weeks)

If other, please specify, e.g. X weeks and Y days.

If the parents request non-resuscitation, what is the highest gestational age at which you would support NOT attempting resuscitation (including intermittent positive pressure ventilation and intubation)? (i.e. if the infant is less premature than this you would support resuscitation against parental wishes).

Please move the circle to indicate gestation.

Not Applicable

20 21 22 23 24 25 26

Lowest gestational

age (weeks)

If other, please specify, e.g. X weeks and Y days.

# Section B O 2 22+3

A mother has gone into extremely premature labour at **22+3 weeks** gestation. She has a dilated cervix and bulging membranes and delivery is thought to be imminent. She has received a full course of antenatal steroids.

She has been counselled and received accurate information about the chance of survival if the infant is born in the next 24 hours, about potential complications of intensive care, and about the risk of long-term neurodevelopmental problems if the infant survives.

If the infant is born and resuscitation is attempted, what is your estimate for the chance of survival without severe disability (to the nearest 5%)?

Not Applicable

0 5 10 15 20 25 30 35 40 45 50 55 60 65 70 75 80 85 90 95 100

Percentage chance

of survival without

severe disability

Please indicate how much you agree or disagree with the following statements.

|  | Strongly agree (1) | Somewhat agree (2) | Neither agree nor disagree (3) | Somewhat disagree (4) | Strongly disagree (5) |
| --- | --- | --- | --- | --- | --- |
| This infant is 'viable' |  |  |  |  |  |
| It is in the infant's best interests to be resuscitated |  |  |  |  |  |

Imagine that the parents are concerned about the risk of death despite treatment, or survival with severe disability. They are requesting termination of pregnancy.

Please indicate how much you agree or disagree with the following statement.

|  | Strongly agree (1) | Somewhat agree (2) | Neither agree nor disagree (3) | Somewhat disagree (4) | Strongly disagree (5) |
| --- | --- | --- | --- | --- | --- |
| This option should be available to them (1) |  |  |  |  |  |

# Section B O 2 23+3

A mother has gone into extremely premature labour at **23+3 weeks** gestation. She has a dilated cervix and bulging membranes and delivery is thought to be imminent. She has received a full course of antenatal steroids.

She has been counselled and received accurate information about the chance of survival if the infant is born in the next 24 hours, about potential complications of intensive care, and about the risk of long-term neurodevelopmental problems if the infant survives.

If the infant is born and resuscitation is attempted, what is your estimate for the chance of survival without severe disability (to the nearest 5%)?

Not Applicable

0 5 10 15 20 25 30 35 40 45 50 55 60 65 70 75 80 85 90 95 100

Percentage chance

of survival without

severe disability

Please indicate how much you agree or disagree with the following statements.

|  | Strongly agree (1) | Somewhat agree (2) | Neither agree nor disagree (3) | Somewhat disagree (4) | Strongly disagree (5) |
| --- | --- | --- | --- | --- | --- |
| This infant is 'viable' |  |  |  |  |  |
| It is in the infant's best interests to be resuscitated |  |  |  |  |  |

Imagine that the parents are concerned about the risk of death despite treatment, or survival with severe disability. They are requesting termination of pregnancy.

Please indicate how much you agree or disagree with the following statement.

|  | Strongly agree (1) | Somewhat agree (2) | Neither agree nor disagree (3) | Somewhat disagree (4) | Strongly disagree (5) |
| --- | --- | --- | --- | --- | --- |
| This option should be available to them (1) |  |  |  |  |  |

# Section B O 24+3

A mother has gone into extremely premature labour at **24+3 weeks** gestation. She has a dilated cervix and bulging membranes and delivery is thought to be imminent. She has received a full course of antenatal steroids.

She has been counselled and received accurate information about the chance of survival if the infant is born in the next 24 hours, about potential complications of intensive care, and about the risk of long-term neurodevelopmental problems if the infant survives.

If the infant is born and resuscitation is attempted, what is your estimate for the chance of survival without severe disability (to the nearest 5%)?

Not Applicable

0 5 10 15 20 25 30 35 40 45 50 55 60 65 70 75 80 85 90 95 100

Percentage chance

of survival without

severe disability

Please indicate how much you agree or disagree with the following statements.

|  | Strongly agree (1) | Somewhat agree (2) | Neither agree nor disagree (3) | Somewhat disagree (4) | Strongly disagree (5) |
| --- | --- | --- | --- | --- | --- |
| This infant is 'viable' |  |  |  |  |  |
| It is in the infant's best interests to be resuscitated |  |  |  |  |  |

Imagine that the parents are concerned about the risk of death despite treatment, or survival with severe disability. They are requesting termination of pregnancy.

Please indicate how much you agree or disagree with the following statement.

|  | Strongly agree (1) | Somewhat agree (2) | Neither agree nor disagree (3) | Somewhat disagree (4) | Strongly disagree (5) |
| --- | --- | --- | --- | --- | --- |
| This option should be available to them (1) |  |  |  |  |  |

# Section B O&N

Medical and professional guidelines relating obstetric and neonatal management often refer to fetuses or infants as “viable” or “non-viable”.

In trying to understand whether a fetus or newborn infant is viable, there are multiple aspects that need to be considered.

We are interested in what you understand by this term.

**Medical intervention:** Thinking about whether or not a fetus or newborn is viable depends on whether the fetus or newborn can survive...

Without medical intervention

With medical interventions that are currently accessible to the infant and the treating team

With medical interventions that could keep the fetus alive, even if they are not accessible to the infant and the treating team (e.g. treatment that is available in a more specialised centre, or in another country) Other

If other, please provide more detail.

**The proportion of infants who survive:** Thinking about whether or not a fetus or newborn is considered viable at a particular gestation depends on whether...

It is possible for infants to survive if born at this gestation

The majority (>50%) of infants born at this gestation will survive

The vast majority (>80%) of infants born at this gestation will survive Other

If other, please provide more detail.

**Survival with or without disability:** Thinking about whether or not a fetus or newborn is considered viable at a particular gestation depends on the proportion of infants that survive...

Without disability

Without severe disability

With or without disability Other

If other, please provide more detail.

Please indicate how much you agree or disagree with the following statements.

|  | Strongly agree (1) | Somewhat agree (2) | Neither agree nor disagree (3) | Somewhat disagree (4) | Strongly disagree (5) |
| --- | --- | --- | --- | --- | --- |
| The gestation at which a fetus/newborn is viable has changed in the last 10 years (1) |  |  |  |  |  |
| Improvements in neonatal intensive care in the last decade change how I feel about resuscitation being offered for infants at 23 weeks gestation (2) |  |  |  |  |  |
| Improvements in neonatal intensive care in the last decade change how I feel about non-resuscitation being offered for infants at 23 weeks gestation (3) |  |  |  |  |  |
| Improvements in neonatal intensive care in the last decade change how I feel about termination of pregnancy being offered at 23 weeks gestation (4) |  |  |  |  |  |
| Laws around termination of pregnancy should change according to the gestation when a fetus is viable (5) |  |  |  |  |  |

**Please consider the following information:**

*Changes in outcomes in NICU have sometimes led to debate about whether there should be changes in the law.*

*In Victoria, the Abortion Law Reform Act 2008 determines doctors’ ability to legally perform termination of pregnancy. At less than 24 weeks gestation a registered medical practitioner may perform a termination. After 24 weeks gestation, a registered medical practitioner may perform a termination only if they, and at least one other medical practitioner, believe termination is appropriate considering current and future medical, physical and social circumstances.*

|  | Strongly agree (1) | Somewhat agree (2) | Neither agree nor disagree (3) | Somewhat disagree (4) | Strongly disagree (5) |
| --- | --- | --- | --- | --- | --- |
| The Abortion Law Reform Act 2008 should change to reflect viability with current techniques for neonatal intensive care used in Victoria (1) |  |  |  |  |  |

# Section C

For the next section of the survey we would like you to consider a potential new medical technique to improve the outcome for extremely premature infants, sometimes referred to as '**ectogestation**'.

We will provide some information about the science behind this technique. We will then ask you to consider what implications you believe this technique should have if it were successful in trials.

*In 2017, a paper in Nature described a technique that involved supporting extremely premature newborn lambs in a liquid environment outside the uterus for a period of up to four weeks (https://www.ncbi.nlm.nih.gov/pubmed/28440792). The lambs were delivered by caesarean section at a level of lung maturity equivalent to ~23 week gestation human infants. Blood vessels in the umbilical cord were connected rapidly to a low-resistance oxygenator circuit, which also provided artificial intravenous nutrition. The lambs were supported within a sealed fluid-filled bag (a “Biobag”), the* *fluid continuously exchanged to prevent infection.*

**For the following questions, please assume that this technique has been evaluated in humans and proven to improve mortality and morbidity rates for extremely premature infants. Please also assume the costs of this technique are equivalent to other forms of neonatal intensive care.**

Imagine that this technique if applied to infants at 22+3 weeks gestation has been

shown to yield 75% survival, with no or mild disability in 75% of surviving infants.

A mother has gone into extremely premature labour at 22+3 weeks gestation. She has a dilated cervix and bulging membranes and delivery is thought to be imminent.

Please indicate how much you agree or disagree with the following statements.

|  | Strongly agree (1) | Somewhat agree (2) | Neither agree nor disagree (3) | Somewhat disagree (4) | Strongly disagree (5) |
| --- | --- | --- | --- | --- | --- |
| This infant is viable (1) |  |  |  |  |  |
| It is in the infant’s best interests to be resuscitated (2) |  |  |  |  |  |

Imagine that the parents are concerned about the risk of death despite treatment, or survival with severe disability. They are requesting non-resuscitation of the infant.

Please indicate how much you agree or disagree with the following statement.

|  | Strongly agree (1) | Somewhat agree (2) | Neither agree nor disagree (3) | Somewhat disagree (4) | Strongly disagree (5) |
| --- | --- | --- | --- | --- | --- |
| I would support this management (ie no “biobag”) (1) |  |  |  |  |  |

Imagine that the parents are concerned about the risk of death despite treatment, or survival with severe disability. They are requesting termination of pregnancy.

Please indicate how much you agree or disagree with the following statement.

|  | Strongly agree (1) | Somewhat agree (2) | Neither agree nor disagree (3) | Somewhat disagree (4) | Strongly disagree (5) |
| --- | --- | --- | --- | --- | --- |
| This option should be available to them (1) |  |  |  |  |  |

**Assume that this technique required caesarean section to be applied successfully.**

A mother has gone into extremely premature labour at 22+3 weeks gestation. She has a dilated cervix and bulging membranes and delivery is thought to be imminent.

Please indicate how much you agree or disagree with the following statements.

|  | Strongly agree (1) | Somewhat agree (2) | Neither agree nor disagree (3) | Somewhat disagree (4) | Strongly disagree (5) |
| --- | --- | --- | --- | --- | --- |
| Caesarean section should be optional at 22 weeks gestation (1) |  |  |  |  |  |
| Caesarean section should be strongly encouraged (2) |  |  |  |  |  |
| Caesarean section should be mandatory (3) |  |  |  |  |  |

Please indicate how much you agree or disagree with the following statements.

|  | Strongly agree (1) | Somewhat agree (2) | Neither agree nor disagree (3) | Somewhat disagree (4) | Strongly disagree (5) |
| --- | --- | --- | --- | --- | --- |
| Ectogestation should become common practice (1) |  |  |  |  |  |
| Ectogestation should not be used to extend viability by treating infants who would not otherwise survive (2) |  |  |  |  |  |
| If ectogestation decreased the gestational age of viability this would influence how I feel about termination of pregnancy being performed at 22 weeks gestation (3) |  |  |  |  |  |
| If ectogestation decreased the gestational age of viability this should influence laws around termination of pregnancy (4) |  |  |  |  |  |

# Section D: Personal Data

Gender

Male

Female

Other

Age (years)

18-30

30-40

40-50

50-60

60-70

70+

What is your professional role?

Consultant

Registrar/ fellow

For how many years have you worked with extremely premature infants?

0-3

3-7

7-15

15+

Do you regard yourself as belonging to any religion?

Yes

No, I'm an atheist

Not sure, I'm agnostic

Display This Question:

If Do you regard yourself as belonging to any religion? = Yes

Which religion do you belong to?

Christianity

Islam

Judaism

Buddhism

Hinduism

Other

Display This Question:

If Do you regard yourself as belonging to any religion? = Yes

How often do you attend religious services?

Never

Once a month or less

Twice a month or more

Display This Question:

If Do you regard yourself as belonging to any religion? = Yes

How important would you say religion is in your life?

Not at all important

Slightly important

Moderately important

Very important

Extremely important

How do you consider your general position towards abortion?

Strongly pro-life

Moderately pro-life

Undecided

Moderately pro-choice

Strongly pro-choice

# Thank you

Please provide any feedback in the text box below, or email the researcher (Dominic Wilkinson: [dominic.wilkinson@philosophy.ox.ac.uk](mailto:dominic.wilkinson@philosophy.ox.ac.uk)).

Please refer to the question numbers if you have any concerns about a particular question. 
Thank you for your help!
