# Supplementary figures and images for: Ectogestation ethics: The implications of artificially extending gestation for viability, newborn resuscitation and abortion
Source: Bioethics. 2019 Nov 7;34(4):371–84. doi: 10.1111/bioe.12682 (PMC7216952; doi:10.1111/bioe.12682)

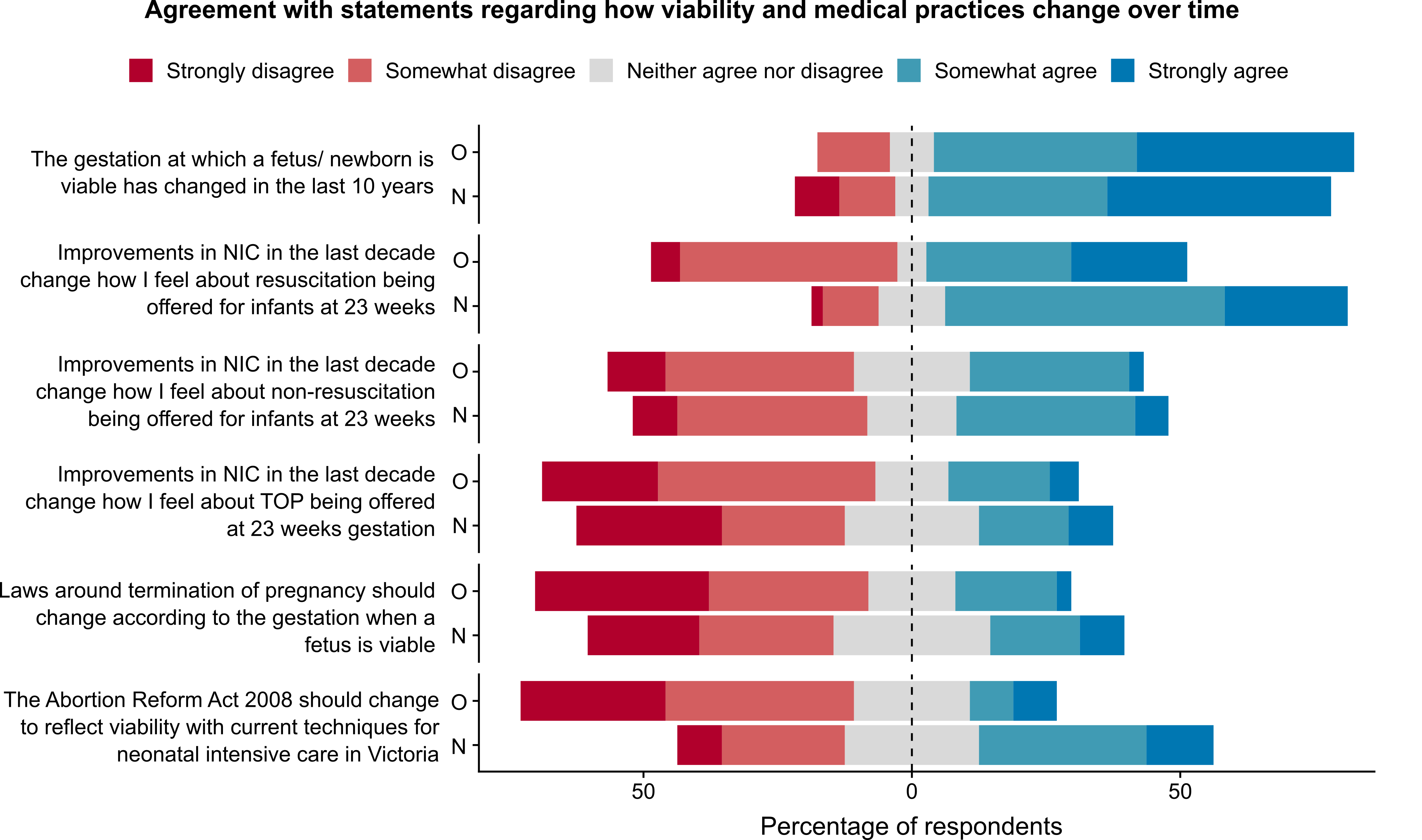

Supplement: Supplementary file 2 [file BIOE-34-371-s002.png]
